# A partnership with the proteasome; the destructive nature of GSK3

**DOI:** 10.1016/j.bcp.2017.10.016

**Published:** 2018-01

**Authors:** Holly Robertson, John D. Hayes, Calum Sutherland

**Affiliations:** a Division of Cancer Research, University of Dundee, Dundee DD1 9SY, Scotland, United Kingdom; b Division of Molecular and Clinical Medicine, University of Dundee, Dundee DD1 9SY, Scotland, United Kingdom

**Keywords:** AMPK, AMP-activated protein kinase, ARE, antioxidant response element, β-TrCP, beta-transducin repeat containing protein, CKI, casein kinase-1, CHD, chromatin helicase DNA-binding factor, CRY, cryptochrome, EMT, epithelial-mesenchymal transition, FGD, faciogenital dysplasia, GSK3, Glycogen Synthase Kinase-3, HECT, homologous to the E6AP carboxyl terminus, JNK, Jun N-terminal kinase, KEAP1, Kelch-like ECH-associated protein-1, LATS, large tumour suppressor, LiCl, Lithium Chloride, LPCAT, LysophosphatidylcholineAcyltransferase, NRF2, nuclear factor-erythroid 2 p45-related factor 2, PAPC, paraxial protocadherin, PI3K, phosphatidylinositol 3 kinase, PPase, phosphatase, PR-A, progesterone receptor-A, PRLr, prolactin receptor, RASSF, Ras association (RalGDS/AF-6) domain family member, REDD1, regulated in development and DNA damage responses-1, RING, really interesting new gene, RTK, receptor tyrosine kinase, SCF, SKP1, cullin 1, F box protein, TAZ, transcriptional co-activator with PDZ, binding motif also known as WWTR1, VEGFR, vascular endothelial growth factor receptor

## Abstract

Glycogen Synthase Kinase-3 (GSK3) was originally reported as a key enzyme of glucose homeostasis through regulation of the rate of glycogen synthesis. It has subsequently been found to influence most cellular processes, including growth, differentiation and death, as part of its role in modulating response to hormonal, nutritional and cellular stress stimuli. More than 100 protein targets for GSK3 have been proposed although only a small fraction of these have been convincingly validated in physiological cell systems. The effects of GSK3 phosphorylation on substrates include alteration of enzyme activity, protein localisation, protein:protein interaction and protein stability. This latter form of regulation of GSK3 substrates is the focus of this review. There is an ever-growing list of GSK3 substrates that upon phosphorylation are targeted to the beta-transducin repeat containing protein (β-TrCP), thereby allowing ubiquitination of bound protein by cullin-1 and so initiating destruction at the proteasome. We propose the existence of a GSK3-β-TrCP ‘destruction hit-list’ that allows co-ordinated removal (or stabilisation) of a set of proteins with a common physiological purpose, through control of GSK3. We identify 29 proteins where there is relatively strong evidence for regulation by a GSK3-β-TrCP axis and note common features of regulation and pathophysiology. Furthermore, we assess the potential of pre-phosphorylation (priming) of these targets (normally a prerequisite for GSK3 recognition) to provide a second layer of regulation delineated by the priming kinase that allows GSK3 to mark them for destruction. Finally, we discuss whether this knowledge improves options for therapeutic intervention.

## Graphical abstract

**Figure:**
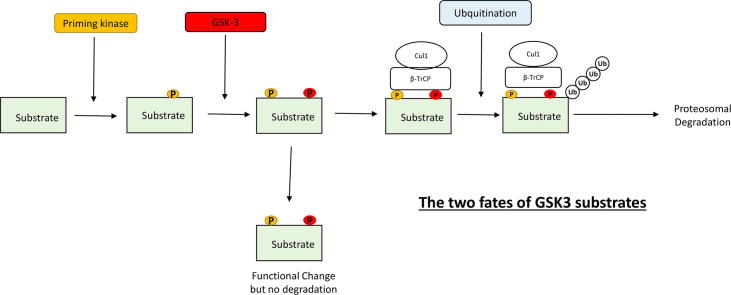

## 1 Introduction

Glycogen synthase kinase-3 (GSK3) is named after the first substrate identified, glycogen synthase, and although it does modulate glycogen synthesis rate in liver and muscle, it is now known to influence many additional cellular processes; including cell proliferation, cell differentiation, neuronal signaling, immune function, inflammation, and nutrient sensing [1], [2], [3]. GSK3 is widely touted as a potential therapeutic target for many chronic diseases. As such, a better understanding of how GSK3 targets its substrates in health and disease could provide a more disease-selective intervention strategy.

## 2 GSK3 biology

### 2.1 GSK3 regulation

There are two mammalian GSK3 genes (GSK3 α and GSK3 β) which are >90% identical in their catalytic domain sequences and which are expressed ubiquitously. GSK3 β deletion results in postnatal lethality, with multiple developmental defects and loss of hepatic function [4]. In contrast, GSK3 α null mice are viable and relatively healthy, with defects in glucose metabolism [5]. Interestingly, GSK3 α null mice have a shorter lifespan than controls and are more prone to chronic age-related diseases [6]. Therefore GSK3 α and GSK3 β isoforms contribute to different non-redundant and vital aspects of healthy ageing in rodents.

GSK3 is central to multiple intracellular pathways including those activated by Wnt/β-catenin, Sonic Hedgehog, Notch, growth factor/RTK, and G protein-coupled receptor signals [7], [8]. It is relatively active in unstimulated cells and mostly regulated by decreasing its activity [3].

There are several regulatory mechanisms which control GSK3 activity; a) phosphorylation of Ser21 in GSK3 α and the equivalent residue in GSK3 β, Ser9 [9], which inhibits phosphorylation of primed substrates [10], b) disruption of GSK3-containing protein complexes such as the axin-APC complex [11], c) phosphorylation of Thr390 of GSK3 β by p38MAPK which inhibits this isoform [12] and d) acetylation [13]. In addition, phosphorylation of Tyr279 of GSK3 α (and the equivalent Tyr216 of GSK3 β) is required for catalytic activity [14], and although this residue is phosphorylated during synthesis of GSK3, this specific modification may be dynamically regulated in neurons [15]. The relative importance of each mechanism to GSK3 regulation may vary from tissue to tissue, and potentially in response to specific stimuli. In particular, growth factors tend to regulate GSK3 through *N-*terminal phosphorylation (although different kinases are used by different stimuli). Meanwhile the canonical Wnt signaling pathway disrupts the interaction between GSK3 and Wnt-specific targets [16], without regulating the *N-*terminal phosphorylation of GSK3 [17]. Therefore, Wnt proteins and growth factors appear to regulate different pools of GSK3 within cells [17] and, as such, these pathways regulate different GSK3 substrates [18]. This is important as changes in total cellular GSK3 activity will not necessarily alter the phosphorylation status of every GSK3 substrate.

### 2.2 GSK3 substrate priming

The majority of GSK3 targets require prior phosphorylation (by a distinct kinase) to generate a GSK3 consensus sequence (SX_3or4_S(P), with the *C-*terminal serine phosphorylated) [17]. This is termed priming and enhances phosphorylation of peptide substrates by GSK3 more than 1000-fold. Priming provides opportunities for physiological, pathophysiological or pharmacological manipulation of specific groups of substrates primed by a common protein kinase, independent of direct GSK3 regulation. That is, physiological or pharmacological inhibition of a priming kinase could reduce GSK3 phosphorylation of only those GSK3 substrates primed by that kinase, even when GSK3 activity was high. The corollary of this is that high GSK3 activity associated with disease does not necessarily mean all GSK3 substrates will be hyperphosphorylated (if priming of some substrates is limiting). Importantly priming of different GSK3 substrates appears to rely on distinct priming kinases. For example, CKI primes APC [19], β-catenin [20], Mdm2 [21], and VHL [22]; CDK5 primes CamKK β [23], CLASP2 [24], CRMP1 [25], CRMP2 [25], Mef2D, and some sites on tau [26]; PKA primes ATP-citrate lyase [27], Ci-155 [28], CREB [29], GATA4 [30] and PP1 G-subunit [31]; ERK2 primes Bcl-3 [32], C/EBP β [33], HSF1 [34], MafA, KRP (telokin) [35], and c-myc/L-myc [36]; DYRK primes eIF2B [37], NFAT [38], tau [26], and possibly CRMP4 [39] and NRF2 (HR unpublished data); and CKII primes glycogen synthase [40], and probably protein phosphatase inhibitor-2 and PTEN [3]. There are at least five additional kinases proposed to act as priming enzymes (including SGK, DNA-PK and AMPK).

### 2.3 Physiology and pathophysiology

The first physiological role that was identified for GSK3 was regulation of glycogen synthesis and glucose metabolism, and this has been studied in great detail for many years [41], [42], [43], [44], [45]. Genetic ablation in mice has demonstrated a vital role for GSK3 β in tissue development [4], while key roles in cell proliferation, cell differentiation, control of microtubule structure, cell cycle progression, and apoptosis, have all been proposed. The diverse range of substrates identified to date would predict that GSK3 has an influence on virtually every cellular process, although it remains to be established how many of these proposed substrates are ‘bona fide’ physiological or pathophysiological substrates [3].

Significant alterations in GSK3 activity have been reported in several age-related human diseases including diabetes, cancer, and Alzheimer’s disease (AD) (see Section 6). Partial deletion (pharmacological or genetic) of GSK3 reduces the development and/or severity of models of these diseases [46], [47], [48] implying a role for GSK3 hyperactivation in their initiation/early progression [3]. As such, several pharmaceutical companies have developed selective, and potent, GSK3 inhibitor small molecules.

At present, there are few data on disease specific substrates of GSK3, the exception possibly being the AD tangle protein, tau [49]. A better understanding of whether it is GSK3 regulation or regulation of specific GSK3 substrates that is altered in human disease could allow a more targeted disease specific intervention. This would likely be more effective and certainly safer than global GSK3 inhibition. For example, phosphorylation of substrates by GSK3 produces a wide range of effects on target function, including altered enzyme activity, protein localization, and protein stability. The list of human diseases associated with both GSK3 defects, and dysregulated protein degradation or accumulation, continues to grow. Therefore, it is likely that validation of the rapidly lengthening list of GSK3 substrates targeted for degradation may provide insight into a mechanistic link between GSK3 and pathophysiology of such diseases.

**Fig. 1:**
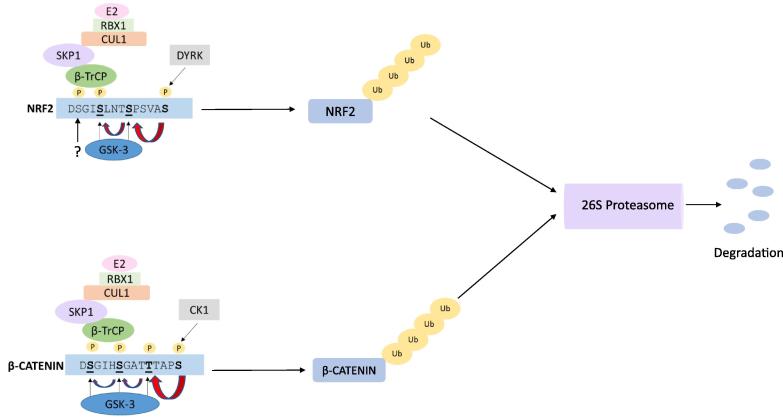
Schematic comparison of two proteins which are targeted for degradation in a GSK3 and β-TrCP-dependent fashion. Pharmacological inhibition of GSK3 activity would be predicted to lower recognition by β-TrCP, preventing ubiquitination by CUL1 and co-ordinated stabilization of both proteins. However there is scope for physiological and pharmacological target specific regulation. 1) β-Catenin, but not NRF2, is present within a complex that permits regulation by Wnt signaling, hence GSK3 inhibition by Wnt signaling would stabilize b-catenin but not NRF2, 2) the two proteins are primed by distinct protein kinases providing the possibility for enhancing or reducing GSK3 targeting through regulation of the priming kinase and 3) GSK3 may only phosphorylate one of the two serines in the NRF2 phosphodegron, and a distinct kinase may be needed to fully engage β-TrCP and enhance degradation, while GSK3 is sufficient to complete the β-TrCP-binding motif in β-catenin.

The remaining sections will investigate the hypothesis that GSK3 is a major regulator of protein stability. In particular we focus on GSK3 substrates that share the same partner for targeting the substrate to the proteasome, namely the SCF (SKP1, cullin-1, F-box protein) E3 ubiquitin ligase β-TrCP. Interestingly, β-TrCP binding of proteins is enhanced by dual phosphorylation of the substrate, with a similar spacing of the two phosphorylated residues to that generated by GSK3 phosphorylation of primed substrates (Fig. 1).

## 3 The GSK3-β-TrCP axis

The ubiquitin proteasome system (UPS) is the predominant route by which eukaryotic proteins are turned over in the cell, and defects in the UPS have been implicated in a range of diseases such as immune disorders, neurological disorders and cancer [50].

### 3.1 Ubiquitination

Ubiquitin is a small (8.5 kDa) ubiquitously expressed protein, encoded by four human genes (UBB, UBC, UBA52 and RPS27A). The covalent attachment of ubiquitin to a target protein (ubiquitination) most commonly results in its degradation by the proteasome.

Ubiquitination involves three basic steps: 1) ATP-dependent activation of ubiquitin, 2) conjugation of ubiquitin to an intermediary protein, and 3) ligation of the ubiquitin to the target protein. These steps are performed by ubiquitin-activating enzymes (E1s), ubiquitin-conjugating enzymes (E2s) and ubiquitin ligases (E3s), respectively. Importantly ubiquitination can involve attachment of a single ubiquitin molecule (mono-ubiquitination) or the generation of a chain of linked ubiquitin molecules (poly-ubiquitination) [51]. The first ubiquitin molecule is covalently bound (ligated) through its *C-*terminal carboxylate group to a particular lysine residue within the target protein, and the chain can then be elongated by linking ubiquitin molecules to one of the seven lysine residues (or the *N-*terminal methionine) of the previous ubiquitin molecule. It is the polyubiquitination on specific lysines, mostly on Lys48 and Lys29 of ubiquitin that leads to degradation of the target protein by the proteasome [52]. Polyubiquitinations at other lysines, or monoubiquitinations, do not normally lead to degradation, rather they can contribute to the regulation of several other cellular processes (including endocytic trafficking, inflammation and DNA repair). This is because only the Lys48 and Lys29 linked polyubiquitin tag can be recognized by the 26S proteasome leading to unfolding of the target protein and ultimately degradation [52].

Some specificity to, and regulation of, this process is provided by the existence of a large family of E3 ubiquitin ligases (>600 members), all with the ability to transfer ubiquitin to the protein target [53]. The E3 ligases can be subdivided into three main groups based on their characteristic domain and their mechanism of action. Firstly, the Homologous to the E6AP carboxyl terminus (HECT) family which form temporary bonds to ubiquitin, before transferring it to the substrate protein. Secondly, the Really interesting new gene (RING) family, which do not bind ubiquitin directly, but serve as adaptors for ubiquitin transfer from the E2 enzymes [54]. Thirdly, the RING-between RING (RBR) family, which contain two RING domains but have a two step mechanism, including the intermediate binding of ubiquitin, as seen in the HECT family.

### 3.2 The SCF (SKP1, cullin-1, F-box protein) E3 ubiquitin ligase family

One of the best examples of the large family of RING E3 ligases is provided by SCF containing complexes, of which there are 69 known in humans [54]. The SCF complex is made up of four components; RBX1, cullin-1, S-phase-kinase-associated-protein 1 (SKP1) and an F-box protein (Fig. 1). Each of these proteins works in concert and provides a unique function to the complex. The RBX1 protein contains the Zinc-binding RING finger domain that enables the transfer of ubiquitin from the E2 ligase to the lysine residue on the target protein, and it also binds cullin-1. The cullin-1 protein functions as a scaffold to link RBX1 to SKP1, while the SKP1 protein brings the F-box protein into the complex. F-box proteins vary in sequence outside their F-box domain and their SKP1 binding site, and this variability provides some target specificity to the complex. This is usually mediated through carboxyl terminal protein:protein interaction motifs, such as WD40 repeats which have a β-propeller structure that recognizes phosphorylated targets, and Leucine-rich repeats (LRR) which have α/β-repeat structures and bind independently of target phosphorylation [55], [56].

β-Transducin repeat containing proteins (β-TrCP, β-TrCP1 and β-TrCP2) are the best studied of the WD40 subfamily of F-box proteins. β-TrCP proteins are responsible for the ubiquitination of a wide range of critical proteins including; IκK phosphorylated IκB, GSK3 phosphorylated β-catenin, cell cycle regulatory proteins CDC25 and APC, and many more [57]. Substrates that are recognized by β-TrCP proteins often contain a destruction motif, DSGXXS, in which X represents any amino acid [57]. Phosphorylation of both serine residues in the motif enhances recognition by β-TrCPs, and thus when phosphorylated, it is sometimes referred to as a phosphodegron.

Misregulation of β-TrCP proteins, has been implicated in cancer development and progression, mainly because it controls the degradation of several tumour suppressor proteins [58]. Relatively high levels of β-TrCP have been detected in cell lines derived from human breast and prostate tumours, and also in breast prostate, and gastric tumour, tissue samples from patients [59].

### 3.3 Interaction between phosphorylation and ubiquitination

As mentioned above, β-TrCP targets often contain a destruction motif, DSGXXS, and the phosphorylation of both of the serines in this sequence greatly enhances the recognition by β-TrCP [60]. As this sequence closely resembles the GSK3 target consensus, (SX_3or4_S(P), with the *C-*terminal serine phosphorylated) it is perhaps not surprising that so many GSK3 substrates are now reported to be β-TrCP binding proteins (Table 1). One of the original GSK3 targets, β-catenin, contains this sequence. It is primed by CK1 for subsequent GSK3 phosphorylation, and this generates a β-TrCP binding motif, thereby enhancing polyubiquitination and degradation by the proteasome (Fig. 1). More recently, another transcription factor, NRF2, was found to be regulated in a highly similar fashion (Fig. 1). Indeed, it seems plausible that GSK3 and β-TrCP co-ordinate the destruction of a wide range of proteins in response to given cues. In the following section (and Table 1) we detail the growing list of GSK3-β-TrCP regulated proteins. It is worth noting that β-TrCP promotes the degradation of many proteins that are not considered GSK3 substrates (eg IkB [61]), thus the GSK3-β-TrCP axis may regulate only a selection of β-TrCP targets.

**Table 1:** GSK3 substrates that are degraded after phosphorylation. Sequences given are human unless specified, the GSK3 phosphorylation site_xxx_ is given in bold and underlined, while the priming site is in bold.

Substrates	GSK3 Phosphorylation site.	Priming Site (Kinase)	GSK3 target Sequence	Ref.
			**Group 1-*Substrates with the DSGX(X)S motif***	
Beta catenin (proto-oncogene)	Ser33, Ser37 and Thr41	Ser45 (CK2)	LD**S**_33_GIH**S**GAT**T**TAPSL	[11], [20], [63]
CHD1 (induces pro-tumourogenic signaling)	Proposed as Ser24 and Ser54	Proposed as Ser28 and Ser58	DD**S**_24_GSA**S**GS and SD**S**54GSE**S**GS	[65]
FGD1 (faciogenital dysplasia) FGD3	Ser283 Ser72	Ser287 (putative) Ser76 (putative)	RD**S**_283_GID**S**ISS RD**S**_7__2_GID**S**PSS	[69], [70]
Prolactin receptor	Ser349	Priming not reported	TD**S**_349_GRGSCDSPSL	[72]
Snail (Triggers EMT)	Ser96 and Ser100	Ser104 (CKIepsilon)	ED**S**_96_GKG**S**QPPSPPS	[66], [67]
Sp1	Ser728 and Ser732	Thr739 (Erks) proposed	LD**S**_728_GAG**S**EGSGTA**T**P	[71]
TAZ (transcriptional co-activator with PDZ-binding motif)	Ser58 and Ser62	Potentially at Ser66	PD**S**_58_GSH**S**RQS**S**TDS	[73], [74]
NRF2	Ser338, Ser342.	Ser347	SDSGI**S**_338_LNT**S**PSVA**S**P(Mouse)	[75], [78]
			**Group 2-*Substrates with a S/TXXXS/T motif*-GSK3 inhibition would enhance cell proliferation.**	
Bcl-3 (b cell lymphoma)	Ser394	Ser398 (ERK)	SPSS**S**_394_PSQ**S**PPRD	[32]
Delta catenin-2	Multiple but Thr1078 proposed	Priming not reported	SSSR**T**_1078_PSISPVRV	[92], [93]
foxp3	Ser270, Ser274.	Priming not reported	LTKA**S**_270_SVA**S**SDKG	[89], [90]
MafA	Ser49, Thr53, Thr57 and Ser61, maybe also Ser65	Ser65 (possible)	LPPG**S**_49_LSS**T**PLS**T**PCS**S**VPS**S**PSFC	[86], [87], [88]
Mcl-1	Ser159	Thr163 (possible-JNK)	STDG**S**_159_LPS**T**PPPAE GADG**S**LPS**T**PPPEEE (mouse)	[82], [83]
Myc	Thr58	Thr62 (ERK2)	ELLP**T**_58_PPL**S**PSRRSG	[84], [85]
Progesterone receptor A	Ser390	Priming not reported	EASQ**S**_390_PQYSFESL	[91]
			**Group 3-*Substrates with a S/TXXXS/T motif*-GSK3 inhibition would reduce cell proliferation.**	
PHLPP1	Ser847 and Thr851	Ser867 and Ser869 (maybe CK1)	PHVQ**S**_847_VLL**T**PQDEFFILGSKGLWD**S**L**S**VEEA	[99], [100]
Smad4	Thr273, Thr269 and Thr265	Thr277 (Erks)	HHNS**T**_265_TTW**T**GSR**T**APYTP	[94], [95], [96]
			**Group 4-*Substrates with a S/TXXXS/T motif*-their GSK3 regulation is related to cellular stress**	
HIF1a	Ser551, Thr555, and Ser589	Priming not reported	KNPF**S**_551_TQD**S**DLDLEMLAPYIPMDDDFQLRSFDQLSPLESSSA**S**PESASP	[101]
LPCAT1 (LysophosphatidylcholineAcyltransferase1)	Ser178	Ser182	FVSR**S**_178_DQD**S**RRKTV	[104]
RASSF1C	Ser19 and Ser23	Priming not reported	STTS**S**_19_GYC**S**QEDSDSE	[103]
REDD1	Ser19, and/or Thr23, and/or Thr25.	Priming for Ser19 may occur at Thr23.	SSPS**S**_19_LPR**T**P**T**PDRPPRS	[102]
			**Group 5-*Substrates with a S/TXXXS/T motif*-their GSK3 regulation is related to development and circadian rhythm**	
Ci-155 (Dros. melanogaster)	Ser852, and Ser884 and 888.	Ser856 (PKA)Ser892 (PKA)	SMQ**S**_852_RRS**S**QSSQVSS DPI**S**_884_PGC**S**RRS**S**QMS **(*Dros. melanogaster sequence*)**	[106], [107]
CRY-2	Ser553	Ser557	RPLP**S**_553_GPA**S**PKRK	[105]
Gli3 (human homologue of ci155)	Ser858, Ser870, and Ser890.	Ser862, Ser874, and Ser894.(all PKA)	AYLS**S**_858_RRS**S**GISPCFS**S**870RRS**S**EASQ STDA**S**_890_RRS**S**EASQ	[108]
PAPC	Ser816 and Ser820	Priming not reported	MGHI**S**_816_TKD**S**GKGD	[109]
			**Group 6-*Substrates that are degraded following phosphorylation by GSK3 but lack a consensus S/TXXXS/T motif***	
Oma1	Thr339	Thr239 proposed	SAGS**T**_339_PSQD (C.elegans sequence) ARPS**T_239_**PDE	[111]
p21cip1	Thr57	Priming not reported	FVTE**T**_57_PLEGDFAW	[110]
			**Group 7-*Substrates whose phosphorylation sequence remains to be determined***	
Securin/PTTG	Sites not identified. Putatively assigned to Ser183/184			[114]
Vegfr-2 (vascular endothelial growth factor receptor)	Not experimentally identified.			[112], [113]

## 4 GSK3 substrate regulation

### 4.1 General

There are over 100 proposed substrates of GSK3 [3], and the proportion of these reported to be degraded in response to GSK3 phosphorylation is growing rapidly ([18] and Table 1). Clearly degradation is the ultimate regulatory mechanism, completely removing all functions of the protein, and raises the interesting possibility that the GSK3-β-TrCP axis is used to co-ordinate the removal of specific sets of proteins in response to different environmental cues. Equally, loss of control of this axis could underpin the observed association of dysregulated GSK3 and its targets in many age-related diseases.

### 4.2 GSK3 and protein degradation

Table 1 lists substrates with compelling evidence that their steady state protein level can be modulated through phosphorylation by GSK3, and subsequent ubiquitination by β-TrCP, or a close homologue. Almost certainly this is not going to represent the final comprehensive list. Indeed, Gumbiner and colleagues performed an expression cloning screen in *Xenopus* eggs which identified 35 novel proteins whose degradation/stability appeared to be regulated by GSK3 inhibition. Of course, it is possible that GSK3 will co-operate with E3 ligases other than β-TrCP, however it seems unlikely to be a coincidence that β-TrCP and GSK3 share such a common recognition motif. That said, there are a few examples of GSK3 targets which go on to be degraded following ubiquitination by Fbw7, fbxl3, skp2 and Smurf1 E3 ligases ([18]). It will be interesting to establish how many of these other E3 ligases exhibit enhanced activity following dual phosphorylation of the target, or whether the phosphorylated residues are not part of the recognition motif. Table 1 mostly focuses on targets where the residues phosphorylated are known and/or the evidence linking GSK3 and β-TrCP activity to degradation is strong. In theory, this table is a draft ‘GSK3-β-TrCP target destruction hit-list’, where the cellular complement of all of these proteins would be reduced by a β-TrCP mediated process following enhanced GSK3 activity. Conversely, their protein levels are likely to increase following physiological or pharmacological inhibition of GSK3. In reality of course, there will be a number of factors determining whether the levels of all of these proteins are regulated by GSK3-β-TrCP in a truly coordinated fashion.

For example, the overall effect of the GSK3-proteasome partnership will be dictated by the affinity for the substrate (phosphorylation rate, steady state phosphorylation, binding to β-TrCP). In addition, the stoichiometry of priming (and the specific priming pathway involved-see Section 5) along with the cellular substrate concentration will influence the proportion of substrate that can be targeted for destruction (very abundant, poorly primed substrates will be depleted very slowly). Each target may also exhibit distinct rates of dephosphorylation, while the expression of target PPases may vary between cell types, or with environmental cues. Finally, the cellular location of the substrate, priming kinase, PPase and ubiquitination machinery may vary with cell type (eg if phosphorylation occurs in a compartment without β-TrCP or other proteasome factors). In this way, one could envision several subgroups of GSK3-β-TrCP targets with distinct rates of destruction dependent on the factors mentioned above. It is likely though that pathophysiological disruption of the GSK3 and/or β-TrCP pathways would produce a global induction of all of the proteins in Table 1, potentially with deleterious consequences to the cell/tissue.

### 4.3 Substrates for the GSK3-β-TrCP axis

The proposed targets of the GSK3-β-TrCP axis can be classified by the phosphodegron sequence that provides the connection between substrate, GSK3 and β-TrCP. We will discuss the proteins in three groups: firstly, those with the proposed ‘perfect’ β-TrCP binding sequence (DSGXXS); secondly, those with a minimal S/TXXXS/T sequence (and these are sub-divided by the functional result of their regulation by GSK3); thirdly, those lacking the dual phosphorylation motif (Table 1).

#### 4.3.1 Substrates with the DSGXXS motif

As mentioned above, the proposed motif for β-TrCP binding is a doubly phosphorylated DSGXXS sequence where X is any amino acid, and as this closely resembles the GSK3 consensus sequence (SX_3or4_S-phos) it seems reasonable to propose that this phosphodegron is central to allowing degradation by a GSK3 and β-TrCP dependent mechanism. One could therefore predict that the presence of the DSGXXS motif within a protein, where phosphorylation occurs at the *C-*terminal Ser, would enhance subsequent phosphorylation at the *N-*terminal Ser by GSK3, binding of β-TrCP, poly-ubiquitination and degradation. Gumbiner and colleagues performed an *in silico* search for protein sequences containing “D/ESGXXS/TXXXS/TXXXS/T”, and identified 38 proteins with this sequence (and with evolutionary conservation) [18]. This list includes β-catenin and snail (which were used to generate the search string) and two other proteins where the stability was already proposed to be regulated by GSK3 or Wnt. The remainder have still to be formally confirmed as GSK3-β-TrCP targets but illustrate the possibility that there are several more GSK3-β-TrCP target proteins remaining to be characterised. Equally, if one considers that only 7 out of the 29 proteins listed in Table 1 contain the precise DSGXXS sequence then there may well be dozens more to be found. It is noteworthy that dysregulation of all 7 of these DSGXXS containing proteins, plus NRF2 (that has a DSGXXS-like motif), are strongly associated with aspects of neoplastic disease, something that is also well established for dysregulated β-TrCP [62].

β-Catenin is a multifunctional protein involved in transcriptional control but also gap junction structure. Intracellular β-catenin is normally sequestered by a destruction complex that includes Axin and APC as well as the protein kinases CK1 and GSK3. CK1 phosphorylates β-catenin within this complex at Ser45, which targets it for sequential GSK3 phosphorylation at Thr41, Ser37 and finally Ser33 [20]. The phosphorylation of Ser37 and Ser33 generates the phosphodegron that binds β-TrCP thereby promoting poly-ubiquitination and proteasomal degradation (Fig. 1). This mechanism regulates the transcriptional activity of β-catenin (not the gap junction structural function) and is regulated by Wnt signaling. Wnt proteins stabilize intracellular β-catenin either by destabilising the complex to separate GSK3 from β-catenin or mono-ubiquitinating GSK3-β-TrCP, both of which have the same outcome of reducing phosphorylation and ubiquitination of β-catenin [11], [63]. The β-catenin is then free to go to the nucleus and regulate target gene transcription. This was the first GSK3 substrate found to be regulated in concert with β-TrCP. Importantly mutations in β-catenin most closely associated with neoplastic disease lie within the phosphodegron sequence, primarily Ser33, Ser37, Thr41 and Ser45 mutated to non-phosphorylatable residues, which would stabilize the β-catenin [64].

The chromatin helicase DNA-binding factor (CHD1) regulates epigenetic modifications of chromatin. Ectopic GSK3 expression resulted in β-TrCP-mediated poly-ubiquitination and degradation of CHD1, while GSK3 inhibition reduced poly-ubiquitination and stabilized CHD1. In normal cells PTEN reduces Akt signaling which reduces the Akt-mediated inhibition of GSK3. This stimulates the GSK3-mediated phosphorylation of two CHD1 phosphodegrons, resulting in CHD1 degradation via the β-TrCP-mediated ubiquitination-proteasome pathway. PTEN deficiency (often found in neoplastic disease) results in hyperactivity of Akt, reduction in GSK3 activity, and stabilization of CHD1, which in turn engages the trimethyl lysine-4 histone H3 modification to activate transcription of the pro-tumorigenic TNF-NF-κB gene network [65]. Thus, CHD1 depletion through inhibition of Akt and re-activation of GSK3 could reduce growth and survival of PTEN-deficient prostate and breast cancers.

Snail (a zinc-finger transcription factor) triggers the epithelial-mesenchymal transition (EMT) during embryonic development through its inhibition of E-cadherin expression [66], [67]. The phenotypic changes of increased motility and invasiveness of cancer cells are reminiscent of the EMT suggesting a possible link between Snail dysfunction and cancer. Snail is one of the EMT factors whose dysregulation is proposed to contribute to the initiation of specific cancers [68]. GSK3 β binds to and phosphorylates Snail at two phosphodegron motifs [66]. Phosphorylation of the first motif (Ser96-Ser100) regulates its β-TrCP-mediated poly-ubiquitination, whereas phosphorylation of the second motif controls its subcellular localization. A variant of Snail which lacks regulation by phosphorylation is much more stable and resides exclusively in the nucleus, thereby inducing EMT. Furthermore, inhibition of GSK3 results in the upregulation of Snail and downregulation of E-cadherin *in vivo* [66]. Thus, Snail and GSK3 may be regulated by many signaling pathways that lead to EMT. Importantly CK1 phosphorylation of Snail at Ser104 is required for the subsequent GSK3 phosphorylation at Ser100 and then Ser96, creating the doubly phosphorylated DSGXXS motif with enhanced binding to β-TrCP [67]. Specific inhibition or depletion of CK1ε inhibits the phosphorylation and degradation of Snail and promotes cell migration, demonstrating that targeting the priming phosphorylation of a GSK3 substrate can alter its regulation by GSK3 without changing the activity of the GSK3-β-TrCP proteins themselves.

FGD1 gene disruption results in Faciogenital Dysplasia (FGDY; Aarskog syndrome), a skeletal dysplasia and multiple congenital anomaly syndrome, implying a crucial role in aspects of mammalian development. The gene product contains domains that indicate it is a guanine nucleotide exchange factor (GEF) for the Rho family GTPase Cdc42. FGD1 contains a DSGXXS motif and is recognized by β-TrCP when the two serine residues in this sequence are phosphorylated. This promotes ubiquitination and subsequent degradation of GEF by the proteasome [69]. The ubiquitination and degradation of GEF is regulated by GSK3 inhibitors. Subsequent work found that FGD3, a homologue of FGD1, is degraded following polyubiquitination by β-TrCP, and this is also regulated by Lithium Chloride (LiCl), a non-selective GSK3 inhibitor [70]. FGD3 has highly homologous domains to FGD1 that suggest it is also likely to be a GEF for Cdc42, as well as having the DSGXXS motif. However, when overexpressed, FGD3 induces distinct cellular phenotypes to FGD1 in HeLa cells, indicating they possess somewhat distinct cellular roles. Despite this, it appears their destruction may be coordinated through the GSK3-β-TrCP axis.

Sp1 is a ubiquitous transcription factor linked to the regulation of dozens of genes. Glucose starvation or treatment with the thiazolidinedione derivative (OSU-CG12) both induce specific binding of Sp1 to β-TrCP followed by Sp1 degradation in LNCaP cells [71]. In addition, ectopic expression of β-TrCP enhances the ability of OSU-CG12 to facilitate Sp1 degradation, while suppression of endogenous β-TrCP function, using either a dominant-negative mutant or small interfering RNA-mediated knockdown, blocks OSU-CG12-facilitated Sp1 ubiquitination and/or degradation. Sp1 contains a C-terminal conventional DSG destruction box ((7 2 7)DSGAGS(7 3 2)) that mediates β-TrCP recognition and this is phosphorylated by GSK3 *in vitro* [71]. Phosphorylation of Thr739 by ERKs appears critical for Sp1 degradation and it may be that this acts as a ‘distant’ priming site for GSK3 phosphorylation of Ser732 and Ser728. Alternatively, there may be a third kinase which is actually responsible for Ser732 phosphorylation *in vivo*, providing priming for GSK3 phosphorylation of Ser728. If so, this would mean there is a requirement for three different kinases to phosphorylate Sp1 before it was shuttled to the proteasome.

The prolactin receptor (PRLr) is a cytokine receptor best known for its role in mediating prolactin control of mammary growth. However, it is expressed in many cell types besides mammary cells, including pancreatic beta cells and adipose tissue, and so it contributes to reproduction, islet differentiation, fat storage and immunomodulation. GSK3 phosphorylates PRLr on Ser349 *in vitro*, and this phosphorylation is required for the recognition of PRLr by β-TrCP, as well as for PRLr ubiquitination and degradation [72]. PRLr is relatively abundant in human breast cancer, potentially due to decreased phosphorylation of Ser349. Constitutive oncogenic signaling downstream of ErbB2 and Ras stabilizes PRLr via inhibitory phosphorylation of GSK3 β on Ser9. Meanwhile progressive inactivation of GSK3 β correlates with elevated levels of PRLr protein in human breast cancer tissue [72]. Interestingly, although the sequence around Ser349 (D**S_349_**GRGS) is a perfect DSGXXS motif there is no report as yet of phosphorylation of Ser353 being required for GSK3 to phosphorylate PRLr or for binding to β-TrCP. One might predict the existence of a priming kinase that initiates the pathway by phosphorylation of Ser353.

TAZ (transcriptional co-activator with PDZ-binding motif; also known as WWTR1) is a transcriptional co-activator mainly controlled by the Hippo pathway. The Hippo pathway is a key regulator of organ homeostasis through modulation of cell proliferation and apoptosis. TAZ is inhibited by large tumour suppressor (LATS)-dependent phosphorylation, leading to cytoplasmic retention and ubiquitin-dependent degradation. The LATS kinase, a core component of the Hippo pathway, phosphorylates a *C-*terminal sequence in TAZ to promote its degradation. However, there is also an *N-*terminal DSGXXS sequence within TAZ that is regulated by PI3K signaling [73]. GSK3 phosphorylates this sequence in TAZ, promoting binding to β-TrCP, ubiquitination and degradation. PI3K signaling inhibits GSK3 thereby reducing phosphorylation of TAZ and stabilizing the transcriptional coactivator. TAZ can also be regulated by Wnts [74], again suggesting that the GSK3-β-TrCP axis controls TAZ stability.

One protein with a DSGXXS-like motif is Nuclear factor-erythroid 2 p45-related factor 2 (NRF2, encoded by the gene *NFE2L2*), a cap‘n’collar (CNC) basic-region leucine zipper (bZIP) transcription factor that mediates intracellular redox homeostasis. It is best known for its activation during oxidative stress, but it is becoming more appreciated for its contribution to other cellular stress responses [75]. NRF2 is activated by oxidative stressors and electrophilic agents, and mediates adaptation to stress by positively regulating cellular antioxidant defences and metabolism through its control of over 250 target genes in response to various stressors. These gene products regulate a host of cytoprotective functions including; drug detoxification, GSH-based antioxidants, Thioredoxin-based antioxidants, pentose phosphate shunt, NADPH generation, purine synthesis, iron sequestration, proteasome subunit production, apoptosis, serine/glycine biosynthesis and autophagy.

In most cell culture conditions NRF2 has a very short half-life primarily due to rapid ubiquitination and 26S mediated proteasomal degradation. However, upon exposure to oxidative stress conditions, or to thiol-reactive compounds, nuclear levels of NRF2 protein increase thereby driving antioxidant response element (ARE)-driven gene expression. Under non-stressed conditions the ubiquitination of NRF2 is predominantly performed by cullin-3 (CUL3) using Kelch-like ECH-associated protein-1 (KEAP1) as a substrate adaptor protein for the CUL3 RING-box 1 (RBX1) E3 ligase complex. KEAP1 functions as a stress sensor, under oxidative stress conditions several key cysteine residues on the surface of KEAP1 are modified. The modified KEAP1 loses its ability to induce cullin-3 ubiquitination of NRF2. As a consequence, the half-life of newly synthesised NRF2 protein is extended and the expression of ARE-driven NRF2-target genes is induced.

Besides KEAP1, NRF2 is also targeted for proteasomal degradation through ubiquitination by cullin-1, using β-TrCP as a substrate adaptor. However, in this instance, ubiquitination of NRF2 by cullin-1 requires the transcription factor to be first phosphorylated by GSK3 in order to generate the phosphodegron to which β-TrCP binds (Fig. 1). In NRF2 peptide pull down experiments, we found that NRF2 possesses two β-TrCP binding sequences within its Neh6 domain [76]. Peptides containing DSAPGS_378_ or DSGIS_338_ (n.b. amino acid numbering based on mouse Nrf2) were able to interact with β-TrCP, with the latter exhibiting much higher affinity if it were doubly phosphorylated [76]. GSK3 could phosphorylate Ser338 (and Ser342) of the Neh6 domain implying that this provides at least part of the modification that enhances β-TrCP binding [76], [77]. Recent work has also demonstrated the requirement for priming of NRF2 at Ser347 in order to initiate the phosphorylation by GSK3 (Fig. 1 and HR and Sudhir Chowdhry unpublished data). NRF2 priming is likely to be mediated by members of the CMGC family of kinases and provides an additional regulatory point for control of NRF2 stability [76], [78]. Inhibition of either GSK3 or the priming kinase would stabilize NRF2.

There are two key differences between NRF2 and the GSK3-β-TrCP targets listed above: firstly, it is a DS335GXS338 motif; secondly, robust evidence that GSK3 directly phosphorylates the *N-*terminal Ser (Ser335) within the β-TrCP binding motif is still lacking. Indeed, the spacing between the Ser residues in the DS335GXS338 is less than three amino acids, indicating it is highly unlikely that GSK3 phosphorylates Ser335 using Ser338 as the ‘priming site’. However, there is strong evidence that GSK3 phosphorylates Ser338 using Ser342 as a priming site [76], and this is crucial to allow β-TrCP-mediated NRF2 degradation. The identity of the kinase that phosphorylates Ser335 remains uncertain.

Interestingly, the related CNC-bZIP transcription factor NRF1 is also degraded by the proteasome in a β-TrCP-dependent manner through a DSGLS motif [79], but in this case the recognition motif is phosphorylated by casein kinase 2, and not GSK3 [80].

#### 4.3.2 Substrates with a minimal S/TXXXS/T motif

The β-TrCP binding motif is clearly more degenerate than the ‘classic’ DSGXXS motif. For example, our work on NRF2 found DSGXS to be a high affinity interaction sequence after dual phosphorylation [76], and additional non-consensus β-TrCP binding sequences, that do not involve phosphorylation have been reported, including in NRF2 [76]. However, recent structural studies have confirmed that dual phosphorylation greatly enhances β-TrCP binding affinity, while it also established that different residues on β-TrCP are involved in binding different phosphorylated substrates [60]. This is consistent with the precise DSGXXS sequence not being prescriptive for β-TrCP targeting, and explains the large number of phosphoproteins in Table 1 that are degraded in a β-TrCP-dependent fashion despite the fact they lack this exact consensus motif. Sixteen of the proteins in Table 1 that have a SXXXS motif (in addition to the 7 with DSGXXS plus NRF2 with DSGXS) exhibit enhanced degradation (in a β-TrCP-dependent manner) when double phosphorylation of this motif is present. Moreover, a further two (Progesterone receptor A and δ-catenin) have the potential to be doubly phosphorylated but as yet are only reported to be phosphorylated on the *N-*terminal residue within the SXXXS sequence (by GSK3). Thus, amongst the 27 proteins in Table 1 where the GSK3 target sequence is known, a total of 25 are likely to share the dual phosphorylation as central to their destruction by the proteasome.

However there doesn’t appear to be any consistent pattern in the primary sequence of the ‘phosphodegron’ (outside the dual phosphorylation sites) to explain recognition of this series of proteins by β-TrCP (Table 1). These proteins only account for around a quarter of the 100 or so proposed GSK3 substrates (almost all of which contain this minimal S/TXXXS/T motif), and the rest do not appear to be poly-ubiquitinated and degraded upon phosphorylation [3]. This means that there must be a structural component, in addition to dual phosphorylation, that dictates whether β-TrCP ubiquitinates the phosphorylated GSK3 substrate.

The simplest explanation is the existence of a scaffold protein(s) that enhances interactions between GSK3, β-TrCP and the substrates for degradation, in an analogous fashion to the canonical Wnt signaling complex [11]. Indeed, the axin scaffold may bring many additional GSK3-β-TrCP targets in close proximity to GSK3 and β-TrCP, and allow their regulation by Wnts [18], [81]. However, there are few reports of β-TrCP or GSK3 containing complexes which could co-ordinate Wnt-independent regulation of GSK3 substrates, although one would predict these may be difficult to isolate as they would be short-lived and their formation dynamically regulated. It is possible that the physiological functions of these substrates may provide clues as to how, and why, they may be co-ordinately regulated. Many of the substrates in Table 1 are involved in cell growth, differentiation and/or cell death, and it may be that they are regulated by a common mechanism related to the control/timing of these processes in order to co-ordinate their destruction.

##### 4.3.2.1 Proteins with an S/TXXXS/T motif whose stability would be increased by GSK3 inhibition with subsequent enhancement of cell proliferation

Mcl-1 is an anti-apoptotic member of the Bcl2 family, that is lost from cells exposed to UV irradiation [82]. Phosphorylation at Thr163 by JNK primes Mcl-1 for phosphorylation at Ser159 by GSK3, generating a phosphodegron recognized by β-TrCP, leading to ubiquitination and degradation of Mcl-1 in response to UV stress. This links the pro-apoptotic activity of JNK, and the pro-survival activity of the Akt pathway that inhibits GSK3 [82]. In addition, phosphorylation of Mcl-1 by GSK3 in response to Akt inhibition also leads to β-TrCP mediated degradation of Mcl-1. Importantly the induction of apoptosis by Akt inhibitor in NSLCS cells required GSK3 activity, providing evidence for GSK3 regulation of Mcl-1 dependent apoptosis [83].

BCL-3 is a proto-oncogene that regulates immune cell growth through its interaction with NF-kB [32]. Certain B cell leukemias are associated with DNA translocation of the *bcl-3* gene. Interestingly phosphorylation of BCL-3 modulates its association with HDAC1, -3, and -6 and attenuates its oncogenicity by selectively controlling the expression of a subset of target genes such as SLPI and Cxcl1 [32]. GSK3-mediated BCL-3 phosphorylation occurs following priming by ERKs and promotes polyubiquitination and degradation. Although degradation requires the generation of the doubly phosphorylated phosphodegron favoured by β-TrCP, confirmation of β-TrCP as the E3 ligase responsible remains to be confirmed.

Myc is a transcription factor that enhances expression of a broad spectrum of genes involved in the biogenesis of nucleotides and ribosomes, thereby stimulating protein translation. In addition, Myc induces the expression of several cyclins and suppresses the transcription of cyclin-dependent kinase inhibitors, thereby promoting exit from quiescence and stimulating progression through G1 phase. GSK3 phosphorylates Thr58 of Myc and this is enhanced by priming at Thr62, probably by ERKs. Initially phosphorylation of Thr58 of Myc by GSK3 was reported to enhance binding of the E3 ligase SCF^fbw7^ leading to polyubiquitination and degradation of Myc, primarily during the G1 phase of the cell cycle [84]. More recently β-TrCP was also shown to bind to and enhance ubiquitination of c-Myc but modifying alternative residues of Myc to SCF^fbw7^ [85]. This had the effect of promoting Myc stability, thus alternative ubiquitination events at the N-terminus of Myc in response to phosphorylation promote opposite effects on its stability. It appears that the GSK3 regulation of c-Myc may be to target it for labelling by more than one Fbox protein, which ubiquitinate on different residues of c-Myc and have contrasting outcomes on protein stability [85].

The Maf oncoproteins (i.e. large Maf proteins, as opposed to small Maf proteins that heterodimerize with CNC-bZIP transcription factors) are transcriptional regulators of the AP-1 superfamily. They regulate developmental, metabolic, and tumorigenic pathways in multiple tissues and are overexpressed in about 50% of human multiple myelomas [86]. MafA controls insulin production in the pancreatic beta cells in response to glucose. Paradoxically, phosphorylation by GSK3 at multiple sites induces MafA oncogenic potential but at the same time leads to MafA ubiquitination and degradation [86], [87], [88]. Low glucose enhances MafA phosphorylation and degradation in Min6 cells, reducing insulin gene transcription. Meanwhile GSK3 inhibitors reduce the phosphorylation and degradation of c-Maf in multiple myeloma cells but also reduce the inherent oncogenicity of c-Maf, as they reduce proliferation of the cells [88]. While phosphorylation by GSK3 results in 4 or 5 phosphorylated residues lying in series with 4 amino acid spacing (S49-T53-T57-S61-*S65*) it has not yet been confirmed that β-TrCP is the E3 ligase responsible for targeting to the proteasome. GSK-3-mediated Maf phosphorylation also modulates extracellular matrix remodeling which could modify cancer progression [86].

Foxp3-expressing Treg cells limit anti-tumour responses and allow the persistence and growth of cancer. Phosphorylation of Foxp3 by GSK3 promotes β-TrCP binding and polyubiquitination leading to degradation by the proteasome. EGF-like growth factor Amphiregulin (AREG), which is frequently upregulated in human cancers, reduces GSK3 activity and stabilizes Foxp3 [89]. Therefore interfering with the regulation of Foxp3 by AREG in cancer patients could lead to Foxp3 protein degradation in Treg cells and provide a potential novel therapeutic target for cancer treatment [89], [90].

Progesterone receptors (PR-A) are over expressed in Brca1-deficient mammary epithelial cells. BRCA1 germ line mutations increase the risk of breast and ovarian cancer, whereas treating with anti-progesterone delays mammary tumorigenesis in Brca1/p53 knock-out mice. All of these data indicate that progesterone has a critical role in initiation and/or progression of breast carcinogenesis. GSK3-mediated phosphorylation of Ser390 in PR-A promotes its ubiquitination and degradation [91]. Although no priming event was reported for Ser390 phosphorylation, a Ser residue at position 394 exists that would lend itself to a priming mechanism. Meanwhile, expression of a PR-A^S390A^ phosphorylation deficient mutant in human breast MCF-10A epithelial cells enhances proliferation and formation of aberrant acini structures in 3D culture. Reduced phosphorylation of Ser390 of PR-A and relatively low GSK3 β activity is found in the Brca1-deficient mammary gland [91]. This is consistent with Brca1 enhancing GSK3 phosphorylation of PR-A and promoting its subsequent degradation. Brca1 deficiency would associate with enhanced PR-A expression and higher sensitivity to progesterone.

δ-Catenin polyubiquitination occurs following phosphorylation at Thr1078 by GSK3 and generation of the phosphodegron which recruits β-TrCP1 [92]. The interaction between δ-catenin and β-TrCP is enhanced by phosphorylation at Thr1078 [93]. No priming has been reported but there is a phosphorylatable residue that is located four residues *C-*terminal to the proposed GSK3 target site. δ-Catenin can be degraded by the proteasome and also by lysosomes. Numerous downstream processes are regulated by δ-catenin, including control of cognitive function, E-cadherin signaling and angiogenesis. Enhanced δ-catenin stability is associated with several neoplastic diseases including those of prostate, lung, ovary, brain and colon.

##### 4.3.2.2 Proteins with an S/TXXXS/T motif whose stability would be increased by GSK3 inhibition with subsequent reduction in cell proliferation

Smad4/DPC4 is an essential transcription factor in the TGF-β pathway and is frequently mutated or deleted in prostate, colorectal, and pancreatic carcinomas. Smad4 activity is directly regulated by the Wnt and fibroblast growth factor (FGF) pathways through hierarchical phosphorylation by GSK3 and Erk. FGF activates ERK, which primes three sequential GSK3 phosphorylations on Smad4 that generate a Wnt-regulated phosphodegron bound by β-TrCP [94]. In *Xenopus* embryos, these Smad4 phosphorylations regulate germ-layer specification and Spemann organizer formation. Human Smad4 mutations that enhance phosphorylation at Thr273/Thr269 and Thr265 are less stable, inactivate TGF-β signaling and are associated with tumourigenesis [95]. This can be corrected with GSK3 inhibition providing a novel therapeutic opportunity in Smad4 related cancer [96]. Interestingly the related proteins Smad1 and Smad3 are also phosphorylated by GSK3 in similar sequences to the Smad4 motif, thereby targeting them for destruction, although the priming for these could be ERKs or Cdk8/9, while ubiquitination appears to be performed by the Smad specific HECT E3 ligases of the Smurf family [97], [98].

PHLPP1 is a member of a family of Ser/Thr protein phosphatases (PPases) that serve as tumour suppressors by negatively regulating Akt signaling and its expression is reduced in colorectal cancer [99]. β-TrCP recognizes PHLPP1 only after phosphorylation of PHLPP1 by CK1 and GSK3, resulting in enhanced degradation via the proteasome [100]. Ectopic expression of a degradation-deficient PHLPP1 mutant in colon cancer cells enhances dephosphorylation of Akt and inhibits cell growth. Therefore, the GSK3-β-TrCP destruction of PHLPP1 enhances Akt activity which would paradoxically promote greater GSK3 inhibition. This suggests the existence of a negative feedback loop involving Akt and GSK3; when Akt is activated it would inhibit GSK3 and stabilize the Akt PPase, thereby turning off the Akt signal and reducing growth.

##### 4.3.2.3 Proteins with the S/TXXXS/T motif whose GSK3-mediated destruction is related to cellular stress

Hif1a is a critical factor that initiates an adaptive and protective transcriptional programme to protect cells against changes in O_2_ tension (in particular hypoxia). Under normal O_2_ tension it is rapidly degraded following polyubiquitination by the E3 ligase, VHL. However, phosphorylation of Hif1a by GSK3 induces ubiquitination and proteasomal degradation, independent of VHL, and this pathway is proposed to underlie regulation of Hif1a action by growth factors, nitric oxide and hormones [101].

REDD1 (regulated in development and DNA damage responses 1) is a hypoxia-inducible factor-1 target gene, with a protein half-life of <15 min. It has a crucial role in inhibiting mammalian target of rapamycin complex 1 (mTORC1) signaling during hypoxic stress through regulation of TSC proteins. Other environmental stresses such as energy stress, glucocorticoid treatment and reactive oxygen species can enhance REDD1 gene transcription. REDD1 is subject to ubiquitin-mediated degradation mediated by β-TrCP, dependent on phosphorylation by GSK3 [102]. REDD1 degradation is crucially required for the restoration of mTOR signaling as cells recover from stress.

RASSF1A [Ras association (RalGDS/AF-6) domain family member 1A] and RASSF1C are two ubiquitously expressed isoforms of the *RASSF1* gene. RASSF1A is implicated in the regulation of apoptosis, microtubule stability and cell cycle arrest. RASSF1C is a very unstable protein that is polyubiquitinated and degraded via the proteasome. RASSF1C degradation is enhanced when cells are exposed to stress signals, such as UV irradiation [103]. Mule, a HECT (homologous with E6-associated protein C-terminus) family E3 ligase ubiquitinates RASSF1C under normal conditions. In contrast both Mule and β-TrCP target RASSF1C degradation in response to UV irradiation and this requires GSK3 phosphorylation of RASSF1C at Ser19 and Ser23 [103]. Therefore, Mule and β-TrCP target RASSF1C for degradation in response to UV irradiation/DNA damage following phosphorylation by GSK3. Priming was not reported for RASSF1C however GSK3 is not regarded as an UV regulated kinase so it remains possible that an unidentified UV dependent priming mechanism exists for recognition by GSK3.

Lysophosphatidylcholine Acyltransferase 1 (LPCAT1) is a lipid synthesizing enzyme key in the production of the bioactive surfactant phospholipid, dipalmitoylphosphatidylcholine (DPPtdCho) and hence plays a vital role in lung physiology. It is a member of the 1-acyl-sn-glycerol-3-phosphate acyltransferase family converting lysophosphatidylcholine to phosphatidylcholine in the presence of acyl-CoA. This process is also important in the synthesis of platelet-activating factor (PAF). Lipopolysaccharide (LPS) treatment of cells causes LPCAT1 degradation, altering the surfactant properties of the lung. Phosphorylation at Ser178 by GSK3 following priming at Ser182 generates the β-TrCP binding site and enhances polyubiquitination and degradation of the protein in response to LPS [104]. Furthermore, elevated expression of LPCAT1 may contribute to the progression of oral squamous cell, prostate, breast, and other human cancers.

##### 4.3.2.4 Proteins which are destroyed by GSK3 mediated-phosphorylation with vital functions in development and circadian rhythm

Cryptochrome 1 (CRY1) and 2 (CRY2) are flavoproteins that act as essential components of the central and peripheral circadian clocks for generation of circadian rhythms in mammals [105]. They are transcriptional repressors of the transcriptional activators CLOCK and BMAL1 and together these proteins generate a negative feedback loop whose timing provides circadian rhythm to mammalian cells. Mouse cryptochrome 2 (mCRY2) protein accumulates overnight and is phosphorylated at Ser557 in liver. Phosphorylation at Ser557 peaks around 4 h prior to peak protein accumulation and allows subsequent phosphorylation at Ser553 by GSK3, resulting in degradation of mCRY2 by the proteasome. The priming kinase involved is not yet identified although Erk can phosphorylate Ser557 *in vitro* [105]. Inhibitory phosphorylation of GSK3 at Ser-9 exhibits a circadian rhythm with a peak when Ser-557 of mCRY2 is highly phosphorylated. The dual phosphorylation of mCry2 is associated with polyubiquitination (although the E3 ligase responsible is yet to be confirmed) and degradation by the proteasome [105]. The control of the half-life of CRY2 protein contributes to the timing of the circadian rhythm.

In Drosophila, Cubitus interruptus (Ci) is a key transcriptional regulator of limb development. The long form (Ci-155) is an activator of gene transcription while a shorter proteolytic fragment is a repressor [106]. Modulation of the relative proportions of each form of Ci co-ordinates different stages of limb development. PKA phosphorylation of Ci-155 on at least two sites primes it for subsequent phosphorylation by GSK3 and ubiquitination by the Drosophila homologue of β-TrCP called slimb [106], [107]. This enhances the proteolytic processing, generating the smaller repressor form of Ci. Hedgehog (Hh) signaling opposes this proteolysis by promoting dephosphorylation of the PKA and GSK3 target sites.

Gli3 is the human homologue of Ci-155, and is the only Gli isoform that can be processed to a repressor. It also regulates limb development under the control of Shh signaling. In a similar fashion to the Drosophila Hh Ci-155 system, PKA phosphorylation of Gli3 on several sites primes it for subsequent phosphorylation by GSK3 and CK1 [108]. This promotes β-TrCP binding and proteolytic processing, generating the smaller repressor form.

Paraxial protocadherin (PAPC) is involved in gastrulation cell movements during early embryogenesis. It is first expressed in the dorsal marginal zone at the early gastrula stage and subsequently restricted to the paraxial mesoderm in Xenopus and zebrafish. PAPC is also regulated at the protein level and is degraded and excluded from the plasma membrane in the axial mesoderm by the late gastrula stage. PAPC cellular location, ubiquitination and stability were all affected by treatment with a GSK3 inhibitor (BIO) or expression of a dnGSK3. These deficits were reversed by introduction of a Ser816Asp/Ser820Asp ‘double phosphorylation mimic’ mutant PAPC [109]. The poly-ubiquitination of PAPC required β-TrCP, but the possible priming of PAPC to allow GSK3 to recognize it as a substrate has not been investigated. The control of PAPC by phosphorylation/ubiquitination is essential for normal Xenopus gastrulation cell movements [109].

#### 4.3.3 GSK3 substrates that are destroyed following phosphorylation but lack a consensus S/TXXXS/T motif, or evidence for β-TrCP binding

There are two proteins in Table 1 that lack the primary sequence around the GSK3 target site to create a dual phosphorylated β-TrCP binding motif yet they are reported to be degraded in response to phosphorylation by GSK3. Specifically, Oma-1 and p21^Cip1^ contain a Glu or Asp three or four residues C-terminal to the phosphorylated serine and thus it is conceivable that the acidic amino acid side chain can partially mimic a phosphorylated serine and enhance phosphorylation by GSK3. This remains to be confirmed and in our experience an acidic residue does not prime peptide substrates to enhance phosphorylation by GSK3 (Sutherland unpublished data). In addition, the evidence that the degradation reported is mediated by β-TrCP, or even by ubiquitination is still lacking.

Regulation of the DNA repair protein p21^Cip1^ by the phosphoinositide 3-kinase (PI3K)/AKT pathway is crucial for the proper control of endothelial cell (EC) proliferation and survival [110]. p21^Cip1^ is a short-lived CDK binding protein with a high proteasomal degradation rate. The PI3K inhibitors LY294002 and wortmannin reduced p21^Cip1^ protein abundance in human umbilical vein EC. GSK3 phosphorylated p21^Cip1^ at Thr57 within the CDK binding domain [110]. Overexpression of GSK3β decreased p21^Cip1^ protein levels in EC, whereas LiCl interfered with p21^Cip1^ degradation and increased p21^Cip1^ protein about 10-fold in EC and cardiac myocytes. This suggests that GSK3 triggers p21^Cip1^ degradation, while stimulation of Akt increases p21^Cip1^ protein levels via inhibitory phosphorylation of GSK3.

Oma1 has mostly been studied in *C. elegans*. Oocyte maturation and fertilization initiates a dynamic and tightly regulated process in which a non-dividing oocyte is transformed into a rapidly dividing embryo. The zinc finger protein, OMA-1 is expressed only in oocytes and 1-cell embryos, and is degraded rapidly after the first mitotic division allowing embryogenesis to proceed normally. The OMA-1 protein is directly phosphorylated at Thr239 by the DYRK kinase MBK-2, and this is required both for OMA-1 function in the 1-cell embryo and its degradation after the first mitosis [111]. Phosphorylation at Thr239 facilitates subsequent phosphorylation of OMA-1 by GSK3, at Thr339 *in vitro*. Phosphorylation at both Thr239 and Thr339 are essential for correctly-timed OMA-1 degradation *in vivo*. Therefore, GSK3 regulation of Oma1 is required for correctly-timed OMA-1 degradation in one cell embryos [111]. However, it is not clear if this occurs in mammalian cells and it represents a very unusual GSK3 substrate, with non-consensus ‘priming’ occurring at a very distant residue, as well as a non-consensus β-TrCP target, lacking the SXXXS motif. Indeed, the evidence that this is a β-TrCP target is also not yet provided.

#### 4.3.4 GSK3 substrates whose target sequence remains to be determined

There are two proteins in Table 1 with an association between GSK3 phosphorylation, β-TrCP ubiquitination and their degradation, yet the phosphorylation site and β-TrCP binding site remain to be verified, namely VEGFR-2 and securin/PTG.

Vascular endothelial growth factor receptor-2 (VEGFR-2) is a key determinant of the angiogenic response and is decreased in diabetic hyperglycemic mice exposed to oxidative stress. A phosphorylated form of VEGFR-2 was reduced in the mice by hyperglycemia and VEGFR-2 protein was reduced in cells directly exposed to oxidative stress. This coincided with co-location of β-TrCP and VEGFR-2, and increases in ubiquitination of VEGFR-2 [112]. This reduction of VEGFR-2 protein in response to ROS was ameliorated by β-TrCP siRNA or treatment with the proteasome inhibitor MG132 or GSK3 activity inhibitors (LiCl and SB216763). These data together support the hypothesis that oxidative stress induces the GSK3-β-TrCP axis to target VEGFR-2 for degradation [112]. β-TrCP also regulates VEGFR-2 degradation in thyroid cancer cells [113].

Securin is a chaperone protein which binds to separase to inhibit premature sister chromatid separation until the onset of anaphase, and which also modulates cell-cycle arrest after UV irradiation. At metaphase-to-anaphase transition, securin is targeted for proteasomal destruction by the anaphase-promoting complex or cyclosome (APC/C), allowing activation of separase. UV radiation induces a marked reduction of securin protein, while GSK3 inhibitors prevent this securin degradation [114]. β-TrCP ubiquitinates securin *in vivo* and is the ligase responsible for securin degradation after UV irradiation. While GSK3 may mediate this degradation of securin, the site(s) phosphorylated by GSK3 is not known [114]. Securin has a proposed unconventional β-TrCP recognition motif (DDAYPE). If this is truly the motif that mediates β-TrCP binding, then it is unclear what role GSK3 phosphorylation would play in the control of securin.

## 5 Potential input from priming

The prior phosphorylation of a substrate at a residue 4 or 5 amino acids C-terminal to the GSK3 target site greatly enhances the rate of phosphorylation by GSK3. This priming occurs on the majority of validated substrates of GSK3 and introduces an opportunity for substrate selective control of GSK3 action, especially where priming is an essential condition for the substrate to be recognized by GSK3 [3]. There are many different priming kinases identified for GSK3 substrates (see Section 2.2), thus increases in GSK3 activity would not necessarily result in similar increases in phosphorylation of each of the primed GSK3 targets (if priming was limiting). Indeed, it follows that distinct groups of GSK3 substrates will exist in cells (defined by the regulation of priming) which will respond to increases in GSK3 activity in different ways. However, this is a neglected aspect of GSK3 function, which is rather surprising given the enormous interest over the past 20 years in GSK3 as a potential target in the clinic. This lack of study may be related to the fact that priming of the original GSK3 substrate, glycogen synthase, is considered constitutive, and thus priming has always been seen as a relatively inert aspect of GSK3 action.

However, of the 27 proteins regulated by the GSK3-β-TrCP axis where the GSK3 site is known there are 25 substrates where priming could exist (Table 1). Yet a priming kinase was only discussed for 10 of these substrates, and in this small group there are 6 different priming kinases proposed. β-Catenin has the same priming kinase as glycogen synthase (i.e. CK2), while CK1 is thought to prime snail, DYRK isoforms may prime NRF2, JNK primes Mcl-1, ERK is proposed to prime Sp1 and Smad4, and PKA primes the ci155 and Gli3 proteins for GSK3 mediated proteolytic processing (Table 1). This means that the rate of ubiquitination and degradation of each of these substrates would be determined in part by the regulation of their specific priming kinases, as well as GSK3, although all substrates should be stabilized by the inhibition of GSK3.

Clearly most of these proposed priming kinases are part of dynamically regulated pathways, and thus it would seem likely that the priming of the GSK3 substrates will also be dynamically regulated. One obvious example of this is Mcl-1 which becomes a GSK3 target in cells exposed to UV irradiation due to activation of a priming event mediated by JNK [82]. Therefore, careful dissection of priming events in all of these GSK3 targets is urgently needed, as it may be possible to moderate priming mechanisms in order to block disease specific actions of hyperactivated GSK3, and provides an opportunity to develop biomarkers of the priming pathway activity. Importantly, there are already selective inhibitors of some of these priming kinases that have undergone clinical trials, and thus safety and pharmacokinetic information may already be available (Table 2).

**Table 2:** Inhibitors of GSK3 and potential priming kinase mentioned in the text or that have undergone testing in clinical trials.

Kinase	Inhibitor	CAS number	Potential specificity problems	Reported Clinical trials
GSK3 (glycogen synthase kinase 3)	CT99021	CAS 252917-06-9	CDK2-CYCLIN A	None
	Valproic acid/Depakene/Valproate Semisodium/Divalproex	CAS 99-66-1/CAS 76584-70-8		NCT00385710 (completion 2010): Phase 2, Treatment of progressive supranuclear palsy NCT01548066 (completion 2012): Phase 2, for the prevention of hair loss NCT00088387(completion 2005): Phase 2, in combination with lithium for Alzheimer’s disease
	LiCl (Lithium Chloride)	CAS 7447-41-8	MNK1 MNK2 SmMLCK PHK CHK1 CHK2 EF2K NEK6 HIPK3 IKK E TBK1 CK2 PRAK MAPKAP-K2	NCT02601859 (completion 2016): Phase 3, for the prevention of Alzheimer’s disease onset NCT01055392 (completion 2009): Phase 2, treatment of Alzheimer’s disease NCT00088387 (completion 2005): Phase 2, to be used alone or in combination with Divalproex for the treatment of Alzheimer’s disease NCT03290963 (estimated end date 2018): Phase 2, to assess the antidepressant effect of lithium in adults receiving Ketamine NCT01096082 (completion 2013): Phase 2/3, –treatment of Spinocerebellar ataxia type 3 NCT00870311 (completion 2004): Phase 4, for the treatment of Bipolar disorder NCT01543724 (completion 2013): Phase 4, for the treatment of Bipolar disorder NCT01096082 (completion 2013): Phase 2/ 3, for the treatment of Spinocerebellar Ataxia Type 3
	Tideglusib/NP031112	CAS 865854-05-3		NCT01049399 (completed 2011): Phase 2, for the treatment of progressive supranuclear palsy (PSP) NCT00948259 (completed 2009): Phase 1 /2, for the treatment of Alzheimer’s disease
	AZD1080 (5)	CAS 612487-72-6	AMPK LCK MSK1	Phase 1 for Alzheimer’s Disease 2006 (discontinued)
	LY2090314	CAS 603288-22-8		NCT01632306 (study terminated): Phase 1/2, in combination with pemetrexad and Carboplatin for the treatment of Metastatic pancreatic cancer NCT01214603 (completion 2012): Phase 2, for the treatment of acute leukaemia
CK2	CX-4945	CAS 10009820-21-6		NCT00891280 (completion 2011) Phase 1: advanced solid tumour cancers, Castlemans disease of multiple myeloma NCT02128282(completion 2018) : phase 1/2, in combination with cisplatin and gemcitabine in patients with Cholangiocarcinoma
	CIGB-300	CAS 1072877-99-6		NCT01639625 (completion 2016) squamous cell carcinoma or adenocarcinoma of the cervical stage IIA and IIB with cisplatin phase 2 NCT01639638 (study terminated 2014) phase 2 / 3, for patients with recurrent and non recurrent genital condyloma
DYRK	Epigallocatechin-3-gallate (EGCG)	CAS 989-51-5		NCT01394796 (completion 2011): Phase 2, for the treatment of Down Syndrome NCT01699711 (completion 2015): Phase 2, for the treatment of Down syndrome
	GSK626616	CAS 1025821–33-3	YAK3	NCT00443170 (completion 2012): Phase 1 for Healthy subjects with Anaemia
CDK5 (cyclin dependent kinase 5)	R-roscovitine (Seliciclib)	CAS 186692-46-6	CDK2 CK1 DYRK1A CDK1	NCT00999401 (completion 2018) Phase 1 for advanced solid tumours NCT00372073 (completion 2012) Phase2: non-small cell lung cancer (NSCLC)
p42/p44 MAPK ERK1/2	GSK1120212 (Trametinib)	CAS 871700-17-3	MEK1	NCT03232892 (completion 2021) Phase2: non-squamous Non-small cell lung carcinoma NCT01958112 (terminated) Phase 2: cervical cancer NCT01553851 (completion 2015) Phase 2: oral cavity squamous cell cancer
p38-MAPKα/β	SCIO-469 (Talmapimod)	CAS 309913-83-5	TNF-α IL-1 COX-2	NCT00043732(completion 2003) Phase 2: evaluation of safety and tolerability in Rheumatoid Arthritis patients receiving Methotrexate NCT00095680 (completion 2006) and NCT00087867(completion 2005) Phase 2: as a monotherapy or in combination with Bortezomib in relapse refractory patients with multiple myeloma NCT00113893 (completion 2007) Phase 2: treatment for myelodysplastic syndromes NCT00508768 (completion 2002) Phase 1: for the treatment of Rheumatoid arthritis NCT00089921(completion 2004) Phase 2: for the treatment of Rheumatoid arthritis

## 6 Perspectives on GSK3 pathways in the development of novel therapeutics

GSK3 has been touted as a therapeutic target ever since it was discovered as a regulator of glucose homeostasis. Indeed several major pharmaceutical companies developed potent and selective small molecule chemical inhibitors which entered preclinical trials for diabetes and dementia [1], [7], [115], [116], [117], [118]. Interestingly the list of human diseases that are associated with GSK3 dysregulation and/or could benefit from GSK3 inhibition includes inflammation [46], cancer [96], myocardial disease [119], Huntington’s disease [120], Parkinson’s disease [121], kidney disease [122], psychological stress [123], amyotrophic lateral sclerosis [124], and even autoimmune disease [125], bacterial infection [126] and muscle wasting [127].

The wide range of GSK3 inhibitors available for pre-clinical studies has been comprehensively reviewed previously [128]. Interestingly, GSK3 inhibitors that have made it to clinical trials have tended to be relatively non-specific (Table 2), and the broad spectrum of physiological and pathophysiological roles for GSK3, combined with the lethality of a GSK3 β knockout mouse model, has severely restricted the development of such GSK3 inhibitors in the clinic. The fear of wide-ranging effects is perhaps not surprising, especially when scanning the huge list of GSK3 substrates [3], or even the relatively selective range of substrates in Table 1 which appear to share the common outcome of degradation following GSK3 phosphorylation. The majority of these play key roles in cell proliferation and survival, or contribute to fundamental development. Worryingly, there are over a dozen substrates in Table 1 where one would predict enhanced cellular proliferation in response to GSK3 inhibition. In contrast hyperactivation of GSK3 could reduce the production of these substrates, and that would reduce their growth activating functions, with some potential therapeutic benefits in tumourigenesis. For example, β-catenin is a proto-oncogene associated with a variety of common cancers (hepatocellular, breast, colorectal, ovarian and lung). CHD1 depletion suppresses cell proliferation, cell survival and tumorigenic potential of PTEN-deficient prostate and breast cancers [65]. Sp1 is highly expressed in several cancers including colorectal and prostate cancer and is related to poor prognosis [129]. Increased TAZ protein is associated with human cancers, such as breast cancer, and TAZ protein is elevated in tumour cells with high PI3K signaling, such as in PTEN mutant cancer cells. PrLr is commonly stabilized in human breast cancer and NRF2 levels are elevated in several neoplastic diseases, most notably in non-small cell lung carcinoma (NSCLC) and lung squamous carcinoma. NRF2 is frequently upregulated in lung cancer as a consequence of somatic mutations in *KEAP1*, *NFE2L2*, or *CUL3*, and associates with increased cell proliferation and resistance to anticancer drugs. In tumour cells in which the KEAP1-CUL3 ubiquitin ligase is unable to repress NRF2, stimulation of GSK3 activity has been found to suppress NRF2, presumably by increasing ubiquitination of the CNC-bZIP transcription factor by β-TrCP-cullin-1. In this circumstance, and in *Keap1^−/−^* mouse embryonic fibroblasts, inhibition of GSK3 by CT99021 increases NRF2 activity [76]. This in turn is likely to further augment cell proliferation and drug resistance. Any increase in NRF2 signaling would provide a pro-survival effect to the tumour by suppressing ROS levels, increasing NADPH and purine levels, inhibiting the recruitment of immune cells and increasing detoxification of common chemotherapeutics such as Cisplatin and Carboplatin. Therefore, on the basis of these data one could propose GSK3 *activators* rather than inhibitors as possible anti-cancer therapeutics.

That said, GSK3 *inhibition* has been proposed as a potential therapeutic option in specific forms of cancer [96], [130], [131], GSK3 activity is reported to reduce the production of at least two tumour suppressors (Smad4 and PHLPP1 in Table 1) and there has been at least one GSK3 inhibitor taken to clinical trial for anti-tumourogenic potential (Table 2). There are also GSK3 substrates whose phosphorylation by GSK3 associates with tumour development, although this could be linked to enhanced priming, promoting enhanced phosphorylation by GSK3 [132]. In addition, there are few, if any, good examples of cell or rodent models exhibiting robust tumour formation in response to GSK3 gene deletion. Indeed, the GSK3 α KO mouse which lacks two of the four GSK3 alleles does not appear to develop tumours, although it exhibits accelerated ageing by other markers [6]. Finally, lithium, a widely used GSK3 inhibitor, is commonly prescribed to tens of thousands of Bipolar disorder patients around the globe, with no reports of increased risk of neoplastic disease [118]. These data indicate that there is not a simple relationship between GSK3 activity and neoplastic disease.

What is very clear is that if one is to develop safe GSK3-based therapeutic approaches for the clinic then it will be important to understand more about GSK3 activity *and* GSK3 substrate phosphorylation status of the diseases being targeted. For example, is there enhanced GSK3 activity (either globally or specific to the diseased tissue), is there only a subset of GSK3 targets dysregulated, and is any GSK3 defect related to enhanced basal GSK3 or loss of GSK3 regulation? This information is key in order to develop patient-based therapeutic options to interfere with GSK3 function in a manner specific to the pathological issue.

To this end, the development of a panel of GSK3 substrate biomarkers would permit more detailed classification of the many ‘GSK3-associated diseases’. This would normally involve the development of robust phosphospecific antibodies to the residues on the GSK3 targets, ideally with a companion non-phospho antibody to provide a ratio of phosphorylated to non-phosphorylated sequence in each case. The subject of this review (the GSK3 mediated destruction of the target) may provide the opportunity to develop tools to classify these diseases by measuring substrate protein abundance, since in the case of the destruction hit-list this would be directly related to the rate of phosphorylation. In this way a more detailed, extended, and quantitative investigation of the ‘GSK3 destruction proteome’ has the potential to provide biomarker panels for the classification of GSK3 associated disease, which could be assessed in an antibody-based or MS-based assay. An elegant *in vitro* expression cloning strategy in cytoplasmic extracts from *Xenopus* identified 35 novel polypeptides whose expression was modified by LiCl, and a subset of these were also regulated by interfering with Wnt regulation of the axin complex [18], [81]. It would be useful to extend this study to mammalian cells, under different growth conditions, and in different disease states, potentially with a more selective GSK3 inhibitor, or siRNA strategies. In this way biomarker tools could be developed to establish the role of GSK3 targets in the disease process, and to provide key information on the efficacy of interventions aimed at GSK3 activity, or which may alter GSK3 activity as part of their mechanism.

In summary, the emergence of the growing list of GSK3 substrates which are labelled by ubiquitination for destruction via the proteasome may provide some mechanistic understanding of why GSK3 activity is linked to so many diseases. At the same time, there may be opportunities to develop disease specific diagnostic and prognostic biomarker arrays, and hence disease specific or even patient specific therapeutic interventions, based on the GSK3 substrate profile of the disease. It remains to be seen just how large the GSK3-β-TrCP hit-list turns out to be, but it should continue to provide important insights into how cells respond to various stimuli to modify their protein compliment.
